# Single-molecule techniques to quantify and genetically characterise persistent HIV

**DOI:** 10.1186/s12977-017-0386-x

**Published:** 2018-01-09

**Authors:** Xiao Qian Wang, Sarah Palmer

**Affiliations:** Centre for Virus Research, The Westmead Institute for Medical Research, The University of Sydney, 176 Hawkesbury Road, Westmead, NSW 2145 Australia

**Keywords:** Persistent HIV-1, Single-molecule assays, Single-copy assay, Single-genome/proviral sequencing, Full-length individual proviral sequencing, Intracellular HIV-1 reservoirs

## Abstract

Antiretroviral therapy effectively suppresses, but does not eradicate HIV-1 infection. Persistent low-level HIV-1 can still be detected in plasma and cellular reservoirs even after years of effective therapy, and cessation of current treatments invariably results in resumption of viral replication. Efforts to eradicate persistent HIV-1 require a comprehensive examination of the quantity and genetic composition of HIV-1 within the plasma and infected cells located in the peripheral blood and tissues throughout the body. Single-molecule techniques, such as the single-copy assay and single-genome/proviral sequencing assays, have been employed to further our understanding of the source and viral dynamics of persistent HIV-1 during long-term effective therapy. The application of the single-copy assay, which quantifies plasma HIV-1 RNA down to a single copy, has revealed that viremia persists in the plasma and CSF after years of effective therapy. This low-level HIV-1 RNA also persists in the plasma following treatment intensification, treatment with latency reversing agents, cancer-related therapy, and bone marrow transplantation. Single-genome/proviral sequencing assays genetically characterise HIV-1 populations after passing through different selective pressures related to cell type, tissue type, compartment, or therapy. The application of these assays has revealed that the intracellular HIV-1 reservoir is stable and mainly located in CD4+ memory T cells. Moreover, this intracellular HIV-1 reservoir is primarily maintained by cellular proliferation due to homeostasis and antigenic stimulation, although cryptic replication may take place in anatomic sites where treatment is sub-optimal. The employment of single-genome/proviral sequencing showed that latency reversing agents broadly activate quiescent proviruses but do not clear the intracellular reservoir. Recently, full-length individual proviral sequencing assays have been developed and the application of these assays has revealed that the majority of intracellular HIV-1 DNA is genetically defective. In addition, the employment of these assays has shown that genetically intact proviruses are unequally distributed in memory T cell subsets during antiretroviral therapy. The application of single-molecule assays has enhanced the understanding of the source and dynamics of persistent HIV-1 in the plasma and cells of HIV-infected individuals. Future studies of the persistent HIV-1 reservoir and new treatment strategies to eradicate persistent virus will benefit from the utilization of these assays.

## Background

The development of antiretroviral therapy (ART) for the treatment of human immunodeficiency virus (HIV-1) remains one of the great triumphs of modern medicine. However, despite its success, this therapy has a number of limitations. Effective therapy requires meticulous life-long adherence, which many HIV-infected patients find challenging. Nearly all treatment regimens are associated with some short-term and long-term toxicity. Moreover, although therapy suppresses viral replication, it does not completely restore health: treated HIV-1 disease is attended by chronic inflammation, persistent T cell dysfunction, and a shorter life expectancy [1]. In addition, ART is expensive and cannot be delivered sustainably to everyone in need. Finally, and very importantly, since HIV-1 DNA persists as an integrated genome in long-lived or slowly-dividing cellular reservoirs, current therapeutic approaches are unlikely to prove curative [2, 3]. In light of these challenges, treatments aimed at HIV-1 eradication stand out as a highly promising avenue to confront and defeat the HIV-1 epidemic [4, 5]. To move forward down the promising path of HIV-1 eradication strategies, it is critically important to identify where and how reservoirs of HIV-1 persist within HIV-infected individuals on ART and the effects of new curative treatment strategies on these reservoirs.

To measure the level and genetic composition of persistent HIV-1 in plasma, cell and tissue reservoirs, single-molecule techniques such as the single-copy assay (SCA) and single-genome/proviral sequencing assays (SGS/SPS) have been employed [6, 7]. SCA has a broad dynamic range (1–10^6^ copies/ml) and a limit of detection down to 1 copy of HIV RNA [7]. Using this assay, it was found that approximately 80% of participants with plasma HIV-1 RNA levels below 50 copies/ml had quantifiable viremia [7, 8]. Notably, this persistent viremia was evident even after seven years of therapy with an overall median HIV-1 RNA level of 3 copies/ml [9] and can result in viral rebound when effective treatment is terminated [10, 11]. Therefore, monitoring the levels of persistent viremia is not only crucial for confirming the continued effectiveness of ART, but also for determining the effectiveness of new curative treatment strategies for eliminating HIV-1.

Identification of the cells that contribute to the latent HIV-1 reservoir and their maintenance during long-term effective therapy is crucial so that these cells can be targeted for HIV-1 eradication. A well-defined reservoir of HIV-1 is memory CD4^+^ T cells, where HIV-1 latency is established when an activated CD4^+^ T cell becomes infected by HIV-1, but transitions to a memory T cell instead of undergoing lytic infection [2, 12–15]. These memory T cells contain integrated HIV-1 proviruses and the repression of transcriptional initiation (due to the chromatin environment and recruitment of histone deacetylases) or post-transcriptional blocks (nuclear export, translation) enable HIV-1 to evade detection and clearance by the immune system [13, 16, 17]. The study of viral reservoirs has largely focused on components of peripheral blood, but recent findings suggest that most infected cells are actually found in tissue sites—such as the spleen, lymph nodes and GALT—where 90% of lymphocytes are located [18–21]. The application of SGS/SPS assays provides a comprehensive understanding of the genetic characteristics and dynamics of persistent HIV-1 across a range of tissues and cells and how new treatments, such as latency reversing agents affect the genetic composition of the intracellular HIV-1 reservoir [22–28].

The amount of replication-competent HIV-1 in activated, resting memory, and memory T cell subsets or the actual size of the latent HIV-1 reservoir, during effective antiretroviral therapy is unclear [29]. The estimate of latently infected cells is 1 replication-competent provirus per 1 million resting memory CD4+ T cells [2, 30, 31]. However, as recently described by Ho and colleagues, the number of replication-competent proviruses in these cells is underestimated and could be 60-fold higher than previously predicted [32]. The design of future HIV-1 curative therapies require a more thorough understanding of the distribution of replication-competent HIV-1, i.e. the latent reservoir, within T cell subsets. The employment of recently developed full-length individual proviral sequencing assays will assist with the identification of the precise cellular location and amount of genetically intact virus that should be targeted by new curative therapies [32–35].

This review will discuss how the application of single-molecule techniques have enhanced our understanding of the level, location, and cellular mechanisms contributing to persistent HIV-1 in the plasma, cells and tissues of HIV-1-infected individuals on effective therapy. In addition, this review will describe how these technologies have been applied to investigate the effectiveness of curative strategies.

## Quantification of persistent HIV-1 RNA using the single-copy assay

In 2003, the original single-copy assay (SCA) was developed to quantify the levels of persistent viremia in the plasma of participants on effective therapy [7]. This assay uses larger plasma sample volumes (7 ml), improved nucleic acid isolation and purification techniques, and RT-PCR to accurately quantify HIV-1 in plasma samples over a broad dynamic range (1–10^6^ copies/ml). The limit of detection down to 1 copy of HIV-1 RNA makes SCA 20–50 times more sensitive than currently approved commercial assays. To control for recovery of HIV-1, each plasma sample is spiked with an internal virion standard derived from an unrelated retrovirus, the replication competent avian sarcoma-leukosis retrovirus vector RCAS BP(A). The employment of SCA revealed that approximately 80% of participants with plasma HIV-1 RNA levels below 50 copies/ml had quantifiable viremia [7, 8]. This persistent viremia was evident in a study of 40 participants even after 7 years of therapy with an overall median HIV-1 RNA level of 3 copies/ml [9]. The level of viremia correlated with pre-therapy plasma HIV-1 RNA, but not with the specific treatment regimen. A nonlinear mixed effects model revealed a biphasic decline in plasma RNA levels occurring over weeks 60–384: an initial phase of decay with a half-life of 39 weeks and a subsequent phase with no perceptible decay. These data suggest there is a continual cellular source of persistent virus which contributes to viral rebound if ART is terminated.

Low-level viremia has been detected in the plasma of elite controllers; HIV-infected individuals who maintain plasma HIV-1 RNA levels < 50 copies/mL in the absence of any treatment [36–38]. Quantification of paired plasma and cerebrospinal fluid (CSF) samples from elite controllers revealed that significantly fewer CSF samples had detectable HIV-1 RNA compared to plasma samples 19 and 54% respectively (p = 0.02) [36]. Studies that measured HIV-1 RNA levels in paired plasma and CSF samples from ART-suppressed HIV-infected participants using SCA revealed that the proportion of samples with measurable HIV-1 RNA was lower in CSF samples (14–17%) compared to plasma samples (57–64%) and the median levels of HIV-1 RNA in the CSF were significantly lower (p ≤ 0.0001) [39]. HIV-1 RNA was quantified in the CSF even after 10 years of effective therapy and correlated to elevated levels of CSF neopterin, a marker for intrathecal immune activation. To date, it is unknown whether the infrequent and lower amounts of HIV-1 RNA in the CSF of participants on effective therapy reflect viral production within the CNS where ART levels can be lower or virion exchange between CSF and the blood.

Studies of persistent virus using a modified SCA have found that plasma viremia decays slowly with time [40, 41]. In a recent study, molecular beacon technology with single-copy detection was used to quantify HIV-1 RNA in plasma and CSF of participants on effective ART experiencing neurocognitive disorders [42]. These studies revealed that 42% of CSF samples from 220 HIV-positive individuals contained HIV-1 RNA persisting for over 7 months in 69% of these participants. This low-level HIV-1 RNA in the CSF correlated with persistent viremia in the plasma and lower concentrations and distribution of ART into the CNS. However, poor neurocognitive performance was associated with lower HIV-1 RNA levels in the CSF and discordance between HIV-1 RNA levels between the CSF and plasma.

## The effects of treatment intensification on persistent viremia

In addition to the persistence of long-lived latently infected cells, low-level viral replication has been proposed as a mechanism that maintains HIV-1 during long-term effective therapy [43, 44]. If on-going replication is contributing to persistent viremia, treatment intensification, the addition of one or more compounds to existing ART, should reduce this residual viremia. However, treatment intensification—for example, the addition of another drug such as raltegravir, to existing ART or treating participants with an intensified therapy of 5 versus 3 drugs– has shown no perceptible change in persistent viremia in individuals receiving the intensified treatment, which suggests that on-going viral replication is not a probable source of persistent viremia [45–49]. In contrast, some studies of treatment intensification revealed that patients had an increase in episomal (unintegrated) HIV-1 DNA and decreased amounts of unspliced HIV-1 RNA in CD4^+^ T-cells isolated from the terminal ileum [50, 51]. The results of these latter studies support the concept that some viral replication can occur despite suppressive HIV-1 therapy.

## The effects of latency reversing agents and cancer-related therapies on persistent viremia

HIV-1 latency is established when an activated CD4^+^ T cell becomes infected by HIV-1 but transitions to a memory T cell carrying an integrated HIV-1 provirus that is transcriptionally silent, thus evading detection and clearance by the immune system [2, 12, 14, 15]. Current research is focused on developing interventions such as latency reversing agents which involve the use of small molecules approved for cancer therapy, including histone deacetylase inhibitors (HDACis), to induce viral transcription in latently infected cells followed by immune mediated clearance of these virus-producing cells [52–55]. The administration of HDACis, including panobinostat, vorinostat, and romidepsin, to HIV-infected persons on effective ART enhanced intracellular HIV transcription and significantly increased cell-associated HIV-1 RNA (CA HIV-1 RNA) consistent with reversing latency [53–55]. In addition, treatment with panobinostat and romidepsin also increased plasma HIV-1 RNA levels, whereas no effect on plasma HIV-1 RNA levels was found during single-dose or multi-dose vorinostat therapy [52–55]. In 2011, disulfiram [bis(diethylthiocarbamoyl)disulfide], a compound used to treat alcoholism, was found to reactivate latent HIV-1 in a cell-based screen [56]. Clinical trials were established to treat HIV-infected individuals on effective ART with multi-dose disulfiram. The administration of disulfiram transiently increased plasma HIV-1 RNA levels in a subset of participants [57, 58], but there was no demonstrable effect on the size of the intracellular latent HIV-1 reservoir after treatment with HDACis or disulfiram.

Additional compounds being developed for treating cancer are being investigated as potential therapies to reduce persistent HIV-1 [59]. The upregulation of immune checkpoint coreceptors, such as programmed death 1 (PD-1) and cytotoxic T-lymphocyte associated protein 4 (CTLA-4), on malignant cells allows them to avoid immune destruction. Antibodies directed against PD-1, CTLA-4 and a ligand of PD-1 called PD-L1 or immune checkpoint inhibitors are being used effectively in cancer immunotherapy to enhance antitumor responses. Due to chronic HIV-1 antigenic stimulation, immune checkpoint coreceptors are upregulated on CD4+ and CD8+ T cells of HIV-1-infected individuals, resulting in T exhaustion and disease progression [60, 61]. Moreover, cells expressing PD-1 are enriched for integrated HIV-1 DNA in the blood and lymph node indicating that PD-1 expressing cells play a role in HIV-1 persistence [60]. The treatment of six HIV-infected individuals on effective ART with an antibody to the PD-1 ligand, anti-PD-L1, enhanced HIV-1 specific T cells but did not affect the levels of persistent viremia [62]. However, the treatment of an HIV-infected individual on ART with melanoma with anti-CTLA-4 (ipilimumab) resulted in an increase in cell-associated HIV-1 RNA and a cyclical decrease in plasma HIV-1 RNA following each treatment with an overall decline from 60 to 5 copies/ml [63]. Cancer-related therapies are being explored for their capacity to enhance latency reversal or promote killing of virus producing cells and several new therapies are in clinical trials [64].

## Memory T cells contain one HIV-1 DNA molecule

Efforts to eradicate HIV-1 require a comprehensive examination of the quantity and genetic composition of HIV-1 within infected cells located in cells and tissues throughout the body. To determine the relationship among proviruses in cells from peripheral blood and tissue compartments, a single-cell sequencing technique was developed which allowed for the examination of individual viral DNA molecules from single cells. The quantification of viral DNA molecules per infected cell and the relatedness of viral DNA sequences to one another, to DNA in other cells, and to contemporaneous plasma virus RNA can also be determined. The application of the single-cell sequencing assay to cells from untreated HIV-infected participants revealed a correlation between viral RNA levels and frequency of intracellular HIV-1 DNA infection [23]. When analyzing the degree of multiple infection of CD4^+^ T-cells in peripheral blood and lymph node tissue it was found that the vast majority (> 90%) of the CD4+ T cells from peripheral blood and lymph node tissue contained only one HIV-1 DNA molecule, implying a limited potential for recombination in virus produced by these cells [23]. This result is in contrast to the generally accepted belief that most HIV-infected cells contain multiple HIV DNA molecules [65, 66]. These studies demonstrated a similar genetic composition of HIV-1 in lymph node tissue, peripheral blood cells and plasma of untreated participants [24]. This finding implies ongoing exchange between these compartments during untreated HIV-1 infection. In these single-cell studies not one HIV-1-infected monocyte was identified indicating that monocytes are not a major reservoir within HIV-1 infected untreated individuals.

## Genetic characterisation of persistent virus in plasma and cells

To determine the source of persistent viremia and the effects of treatment initiation on the latent HIV-1 reservoir, the genetic composition of persistent virus in the plasma and cells from patients on long-term effective therapy must be assessed. In a seminal study of memory T cell subsets, Chomont et al. found integrated HIV-1 DNA in central memory T cells (T_CM_) and transitional memory T cells (T_TM_). They found that the low proliferation rate of T_CM_ allows them to persist in HIV-1-infected participants with relatively high CD4+ T cell counts. In participants with low CD4+ counts, T_TM_ cells appear to be the major reservoir, which is maintained by IL-7 induced homeostatic proliferation and plasma levels of IL-7 correlated inversely to the rate of decrease of the reservoir. This study suggests that there are at least two cellular mechanisms by which the reservoir within HIV-1-infected memory CD4+ T-cells is maintained [12].

The study of viral reservoirs has largely focused on components of peripheral blood. However, recent findings suggest that most infected cells are actually found in tissue sites—such as the spleen, lymph nodes and GALT—where 90% of lymphocytes are located [18–21, 67, 68]. Therefore, a more comprehensive understanding of the genetic characteristics and dynamics of persistent HIV-1 across a range of tissues and cells is necessary. Single-genome/proviral sequencing (SGS/SPS) has been applied to assess the genetic composition of plasma-derived HIV-1 RNA, cell-associated (CA) HIV-1 RNA and HIV-1 DNA. In conducting these assays, HIV-1 RNA is extracted from plasma and CA HIV-1 RNA and DNA is extracted from cells derived from peripheral blood, gut-associated lymphoid tissue (GALT), lymph nodes, and bone marrow [20–23, 25, 67] and subsequently sequenced at limiting dilution to assess genetic diversity, genetic evolution, and infection frequency [22–25, 27, 69]. Genetic characterisation of HIV-1 DNA extracted from memory T cell subsets including TCM, TTM, effector memory T cells (TEM), and myeloid cells from peripheral blood, GALT, and lymph nodes, from ART-suppressed participants strongly suggests that the primary barrier to a cure is the remarkably stable pool of memory T cells. In agreement with earlier studies, SGS/SPS analyses revealed that naïve T cells contain HIV-1, albeit at a lower infection frequency compared to memory T cell subsets [70–72]. These studies also revealed that participants treated during acute infection had genetically homogeneous HIV-1 populations in all cells from all anatomic compartments and substantially lower HIV-1 reservoir size in blood, gut, and lymph node.

A study by Carter et al. [73] has shown that HIV-1 infects multipotent hematopoietic progenitor cells (HPCs) and that latent HIV-1 infection was established in some of these HPCs, although further research was needed to test whether persistent virus in memory T cells in participants on effective therapy was partially derived from HPCs. Recent studies of HPCs (Lin-CD34-) sorted from bone marrow revealed that these cells do not appear to contain HIV-1 and if this cellular population is infected, the frequency of infection is very low (< 0.0005%) [22, 74]. In addition, studies attempted to investigate the infection frequency of myeloid cells using SGS/SPS, but there was a high likelihood that the sorted myeloid cell population was contaminated with T cells, which still leaves the role of myeloid cells in the persistence of HIV-1 an open question [25, 27]. However, the overall low HIV-1 infection frequency of myeloid cells indicates that if myeloid cells from peripheral blood, GALT and lymph node are infected, their importance as a latent HIV-1 reservoir in participants on ART may be limited.

Finally, applying these sensitive SGS/SPS techniques to compare the genetic composition of intracellular HIV-1 populations to pre-ART plasma extracellular viral RNA showed very low levels of genetic change during long-term effective therapy. In fact, one study estimated that the evolutionary rate was no greater than 0.0006 and 0.002 nucleotide substitutions/site during the 4–12 years of suppressive therapy for the participants treated during early and chronic infection, respectively [25]. These results suggest viral replication is not a major cause of persistence in the cellular populations analysed and that persistent intracellular HIV-1 DNA is most likely maintained by homeostatic and/or antigen-specific cellular proliferation [12, 25–27].

## Anti-latency compounds broadly activate latent HIV-1 proviruses

A promising HIV-1 curative strategy called “shock and kill” involves treating patients on effective antiretroviral therapy with anti-latency compounds, such as histone deacetylase inhibitors (HDACIs), which enhance HIV-1 transcription and reactivate or “shock” provirus from latent reservoirs [52–55]. The administration of the HDACIs, panobinostat, vorinostat, and romidepsin to HIV-1 infected individuals on antiretroviral therapy induces a significant increase in CA HIV-1 RNA from CD4+ T cells [53–55]. However, it is important to discern whether the increases in CA HIV-1 RNA are due to activation of a subset of proviruses or to global non-selective activation of a broad spectrum of latent proviruses. SGS/SPS analyses of CA HIV-1 RNA and DNA and plasma-derived RNA showed that the transcriptomes following panobinostat, vorinostat, and romidepsin administration are genetically diverse and intermingle on phylogenetic trees with intracellular HIV-1 DNA, indicating activation of transcription from an extensive range of integrated latent proviruses [69, 75]. HIV-1 sequences from blood CD4+ T cells and intestinal lamina propria mononuclear cells (LPMCs) of ART-suppressed individuals during and after treatment with panobinostat or romidepsin were compared to sequences from analytical treatment interruption (ATI) plasma after all therapy was stopped. These studies identified CA HIV-1 RNA and DNA sequences in the blood and LPMCs collected during panobinostat or romidepsin treatment that were closely related or identical to plasma sequences from the ATI [69, 75]. This demonstrates that both the intestines and blood are important reservoirs of HIV-1 during effective therapy and that these anatomic sites can harbour HIV-1 capable of emerging during a treatment interruption.

## Full-length individual proviral sequencing to identify the latent HIV-1 reservoir

**Fig. 1:**
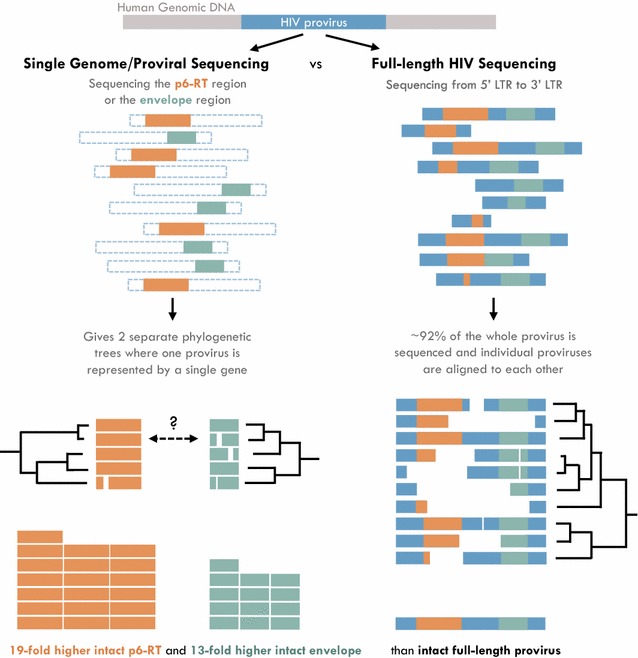
Single-genome/proviral sequencing overestimates the amount of replication-competent proviruses. p6-RT region shown in orange and V1–V3 env region shown in green

The design of future HIV-1 curative therapies require a more thorough understanding of the distribution of replication-competent HIV-1, i.e. the latent reservoir, within T cell subsets. Even though SGS/SPS can provide an in-depth genetic analysis and infection frequency of HIV-1 within specific T cell subsets, these assays overestimate the amount of replication-competent virus which resides within cells (Fig. 1) [34]. Therefore, full-length HIV sequencing of > 90% of the HIV genome has been developed by several research groups [32–35]. The initial full-length HIV-1 assay involved the amplification of four overlapping segments of a single HIV-1 genome which were then sequenced and consolidated into one genome [32]. This assay allowed for the identification of defective versus intact HIV-1 genomes and studies with this method revealed that the latent HIV-1 reservoir was underestimated by earlier in vitro assays [32]. Recently two research groups have developed assays utilizing next generation sequencing to amplify and sequence single near full-length HIV-1 proviruses within CD4+ T cell subsets [34, 35], which allows for in-depth genome-scale analyses of the HIV-1 populations in cells sorted from the peripheral blood and anatomic tissue sites. The application of full-length individual proviral sequencing reveals that intact proviruses which potentially contribute to viral rebound following a treatment interruption were unequally distributed across T cell subsets. In addition, the presence of identical sequence expansions of intact proviruses indicates that proliferating cells contain virus capable of rebound and actively contribute to the latent reservoir.

## Alternative methods for measuring persistent HIV-1

Several methods for measuring persistent HIV-1 have been developed, and these assays identify replication-competent proviruses to varying degrees of sensitivity and specificity (Table 1). These can be categorised into cell culture based assays and PCR-based assays.

**Table 1:** A comparison of the strengths and weaknesses of cell culture and PCR-based assays for the quantification of the HIV-1 reservoir

Assay name	PCR or cell culture based	Strengths of the assay	Weaknesses of the assay
Quantification of HIV-1 RNA in plasma and CSF	PCR	Fast and high throughput	The low levels of viremia in participants on long-term ART could affect accuracy of this assay. Not a true representation of the intracellular reservoir
Quantification of intracellular HIV-1 RNA and DNA	PCR	Fast and high throughput	Overestimates the size of the reservoir. Does not provide an indication of replication competency
Single-genome sequencing	PCR	High throughput	Overestimates the size of the reservoir. Does not provide an indication of replication competency
Full-length individual proviral sequencing	PCR	Relatively high throughput	Expensive technique. Slightly overestimates the size of the reservoir. Replication competency of genetically intact proviruses will require confirmation by in vitro assays
Quantitative viral outgrowth assay	Cell culture	Quantifies replication-competent virus	Requires large numbers of resting memory T cells and is labour-intensive. Underestimates the size of the reservoir due to non-induced proviruses
Tat/rev induced limiting dilution assay	Cell culture	Gives an indication of the size of the inducible reservoir	Requires sizeable numbers of cells and cannot be used for sorted T cell subsets. Overestimates the size of the reservoir

### Cell culture based assays

The principal assay for estimating the amount of replication-competent provirus in resting memory T cells is the Quantitative Viral Outgrowth Assay (QVOA). In performing this assay resting memory CD4+ T cells are cultured at limiting dilution and stimulated with a T cell mitogen, such as phytohemagglutinin (PHA), to activate the transcription of the proviruses within these cells [76]. These activated cells are co-cultured with HIV-1 negative donor CD4^+^ T cells and virions released into the supernatant are then quantified by real-time quantitative PCR or enzyme-linked immunosorbent assay. However, recent studies have shown that not all replication-competent virus is induced by this method and that the QVOA underestimates the HIV-1 latent reservoir by as much as 60-fold [31]. Another assay, the Tat/Rev Induced Limiting Dilution Assay (TILDA) also involves stimulation of enriched CD4+ T cells with PHA and ionomycin to induce provirus expression. These cells are serially diluted, subjected to a pre-amplification RT-PCR step, and then quantified by real-time quantitative PCR using primers and probes specific for the *tat/rev* region [77]. As such, this assay measures the frequency of inducible multiply-spliced HIV-1 RNA in latently-infected cells. Although this assay is more sensitive in detecting the functional HIV-1 reservoir than PCR-based assays (described below), some cells that generate a positive TILDA signal will not produce infectious virions and this can lead to an overestimation of the latent and replication-competent HIV-1 reservoir.

### PCR-based assays

Due to the laborious nature of cell culture assays, as well as the large volumes of sample required, PCR-based assays have been employed as a high-throughput alternative for the quantification of intracellular HIV RNA and DNA [12, 78–80]. These assays amplify intracellular HIV RNA and DNA which is then quantified by real-time PCR or digital droplet PCR techniques [81–83]. However, these PCR-based assays overestimate the size of the viral reservoir as they typically quantify a portion of the HIV genome, such as the LTR region, which gives no indication as to whether the virus is replication competent. The virus could be defective outside of the genomic region which is quantified by these assays.

Single-genome sequencing of a specific viral genomic region provides some insight into the replication competency of a particular provirus, but many proviruses have large internal deletions or defects in genes outside of the sequenced region that will not be detected by this method [34]. Full-length individual proviral sequencing provides a stringent approach to identifying genetically intact HIV-1 proviruses without requiring these proviruses to be reactivated. However, it would require in vitro studies to confirm that the genetically intact proviruses identified by this method are truly replication competent.

## Conclusion

Single-molecule techniques, such as the single-copy assay and single-genome/proviral sequencing assays, have been employed to investigate the source and viral dynamics of persistent HIV-1 during long-term effective therapy. These assays have been employed to determine the effectiveness of new therapeutic treatments in reducing viremia and activating latent virus. Although great strides have been made with these techniques, there are many aspects of HIV-1 persistence that have yet to be explored, such as whether there is on-going replication in anatomic sites where treatment is sub-optimal [84]. Moreover, additional studies are required to fully determine all the cells and anatomic sites where genetically intact replication-competent virus resides.

Looking ahead, the full-length individual proviral sequencing assay holds particular promise to help answer these and other questions concerning the source and dynamics of replication-competent virus. In particular, this assay should be widely applied to interrogate cells from anatomical sites, such as the spleen, liver and central nervous system. This current full-length proviral sequencing assay will need to be complemented with a newly developed full-length HIV-1 RNA sequencing assay in order to provide the fullest possible picture of the latent HIV reservoir and the effects of new curative treatment strategies.

## Abbreviations

ART: antiretroviral therapy
HIV-1: human immunodeficiency virus
SCA: single-copy assay
SGS/SPS: single-genome/proviral sequencing assays
CSF: cerebrospinal fluid
HDACis: histone deacetylase inhibitors
Disulfiram: (bis(diethylthiocarbamoyl)disulfide)
PD-1: programmed death 1
CTLA-4: cytotoxic T-lymphocyte associated protein 4
TCM: central memory T cells
TTM: transitional memory T cells
CA: cell-associated
GALT: gut-associated lymphoid tissue
TEM: effector memory T cells
HPCs: hematopoietic progenitor cells
LPMCs: intestinal lamina propria mononuclear cells
ATI: analytical treatment interruption
PHA: phytohemagglutinin
